# A Dosimetric Comparison of Coplanar Versus Noncoplanar Volumetric Modulated Arc Therapy (VMAT) for High Grade Glioma

**DOI:** 10.7759/cureus.94324

**Published:** 2025-10-11

**Authors:** Sagar Raut, Rajesh Pasricha, Manoj Gupta, Deepa Joseph, Sweety Gupta, Rachit Ahuja, Laxman Pandey, Aathira T S, Nidhi Sharma, Debanjan Sikdar

**Affiliations:** 1 Radiation Oncology, Postgraduate Institute of Medical Education &amp; Research (PGIMER) Satellite Centre, Sangrur, IND; 2 Radiation Oncology, All India Institute of Medical Sciences (AIIMS), Bhopal, IND; 3 Radiation Oncology, All India Institute of Medical Sciences (AIIMS), Rishikesh, IND; 4 Radiation Oncology, Shri Mahant Indiresh Hospital, Dehradun, IND; 5 Radiation Oncology, Rohilkhand Medical College and Hospital, Bareilly, IND; 6 Radiation Oncology, Max Cancer Hospital, Saket, New Delhi, IND; 7 Radiation Oncology, Vardhman Mahavir Medical College and Safdarjung Hospital, New Delhi, IND; 8 Radiation Oncology, BLK Superspeciality Hospital, New Delhi, IND

**Keywords:** coplanar, dosimetry plan, glioma, non-coplanar, vmat

## Abstract

Objective: This study aims to compare the dosimetric differences in radiotherapy plans generated by coplanar volumetric-modulated arc therapy (cVMAT) and noncoplanar volumetric-modulated arc therapy (NcVMAT) for high-grade gliomas.

Material and methods: This is a single-institutional, prospective, observational study conducted during a period of one year from December 2018 to October 2019. A total of 15 consecutive patients with high-grade gliomas who planned to receive postoperative radiotherapy were included in the study. Plans were generated using both cVMAT and NcVMAT for each patient. The plans were evaluated per the International Commission on Radiation Units and Measurements (ICRU) Report 83 criteria. A dosimetric comparison of each plan was made in terms of target coverage and dose to organs at risk (OARs).

Results: On analyzing the results, the two techniques resulted in comparable maximum, mean, and minimum planning target volume (PTV) doses. There were statistically insignificant numerical differences observed in the conformity index (CI) and heterogeneity index (HI). Maximum optic chiasma and ipsilateral cochlea doses were statistically lower in the NcVMAT plan compared to the cVMAT plan (p-value = 0.002). The NcVMAT plans had insignificant differences in monitor units (MU) per fraction.

Conclusion: This study highlights equal PTV coverage, homogeneity, and conformity indices between cVMAT and NcVMAT plans. A better dosimetric profile of the optic chiasma, cochlea, and equal MU per fraction favors the NcVMAT technique for treating high-grade gliomas.

## Introduction

Gliomas are the most common histology of primary central nervous system (CNS) tumors. They are divided broadly as high-grade and low-grade. Of these, glioblastomas are the most common in occurrence and have a dismal prognosis despite maximal surgery and radiation [1,2]. Currently, the treatment of choice for high-grade gliomas is maximal safe resection followed by chemoradiation with adjuvant temozolomide [3]. Though multimodality treatment has resulted in modest survival improvement in CNS tumors, there is a need to improve treatment modalities to minimize acute and long-term treatment-related complications.

Intensity-modulated radiotherapy (IMRT) is a form of conformal radiotherapy wherein the radiation dose is conformed to the target, and exposure of healthy tissues is avoided or reduced [4]. Volumetric-modulated arc therapy (VMAT) is a form of IMRT in which both beam modulation and the shape of the radiation beam can be changed while the gantry is rotating. The advantages of VMAT include reduced treatment delivery time and monitor units; moreover, the target coverage is equal to or better than that of IMRT [5]. Also, VMAT can generate precise conformal dose distribution through rotational delivery accompanied by the variability of the multi-leaf collimator (MLC) position, dose rate, and gantry rotation velocity, thereby improving the dose distribution, reducing the dose to normal tissues, and shortening the delivery time [4-6].

Noncoplanar beams provide an additional degree of freedom and potentially an additional gain in quality of treatment. Noncoplanar arcs are usually used to improve planning target volume (PTV) conformity and homogeneity [7]. While the use of noncoplanar beam arrangements for IMRT, and in particular high-fraction stereotactic radiosurgery (SRS), is common, noncoplanar beam trajectories for VMAT are less common because they require the combination of both gantry rotation and couch rotation, which allows for continuous patient movement.

Though the use of noncoplanar beams in IMRT and VMAT for CNS tumors has been investigated [6,8,9], few studies have compared the dosimetric difference between noncoplanar (NcVMAT) and coplanar VMAT (cVMAT) techniques [9,10]. We conducted this study to evaluate and compare dose distributions to the PTV and organs at risk (OAR) obtained with cVMAT and NcVMAT treatment techniques.

## Materials and methods

This observational study, conducted between December 2018 and October 2019, was approved by the Institutional Review Board of All India Institute of Medical Sciences (AIIMS) Rishikesh (approval number: AIIMS/IEC/18/574). The study included 15 consecutive patients with histologically proven high-grade glioma who had not received any prior radiation therapy to the brain. Written informed con­sent was obtained from all patients. Patients having contraindications for MRI/gadolinium or with diffuse glioma were excluded from the study.

Radiation planning

All patients were immobilized with a head-specific thermoplastic mask. Radiotherapy planning scans (RTP) with a slice thickness of 1.5 mm to 2.5 mm were obtained from the vertex to the root of the neck in treatment position on a dedicated 16-slice CT scanner (DiscoveryTM RT; GE Healthcare, Milwaukee, WI, USA). Scans were imported into the treatment planning system (TPS) (Monaco V5.11.02; Elekta Medical System, Stockholm, SWE). All patients also underwent contrast planning MRI with a slice thickness of 1.5 mm to 2 mm after a gadolinium injection of 0.2 ml/kg covering the whole skull with a 1.5/3 Tesla unit, T1 contrast, T2, and fluid-attenuated inversion recovery (FLAIR) sequences. The MRIs were then co-registered with simulation CT images using the image registration software of the TPS, and the quality of fusion was verified. A single-plan technique was utilized by following the European Organization for Research and Treatment of Cancer (EORTC) 26981_22981 protocol specifications [11]. The gross tumor volume (GTV) included the surgical resection cavity plus any residual enhancing tumor (post-contrast T1-weighted MRI scans). The clinical target volume (CTV) was obtained by expanding the GTV with a 2 cm margin and subtracting from anatomical barriers. A 3 mm isotropic margin was added to CTV for generating PTV.

The prescribed dose was 60 Gy in 30 fractions, in a five-fraction-per-week schedule. The planning criteria set was 95% coverage with 95% of the prescribed dose, while the maximum dose within the PTV doesn't exceed 110%. The OAR that were contoured include the brainstem, optic chiasm, ipsilateral and contralateral eyeballs, lens, optic nerves, cochlea, temporal lobes, and the whole brain minus PTV. The OAR dose constraints were as per the Quantitative Analyses of Normal Tissue Effects in the Clinic (QUANTEC) [12]. Point doses (maximum dose (Dmax) referred to 0.001% of volume and minimum dose (Dmin)) and mean doses (Dmean) were recorded.

Two treatment plans, cVMAT and NcVMAT, using two to four arcs, were generated for all patients using the inverse-planning method. Depending on the location and feasibility, partial or full arcs were used. In the NcVMAT plans, the isocentric arrangement of arcs involved careful placement of the isocenter inside the target to optimize both dosimetric outcomes and mechanical clearance during delivery.

Data collection

Plan evaluation was done using International Commission on Radiation Units and Measurements Report 83 (ICRU-83) [13], which included verification and analysis of the homogeneity index, conformity index, maximum dose, minimum dose, and mean dose in the target. Homogeneity index (HI) was calculated using [14] HI = D95/D5, where D95 is the minimum dose in 95% of PTV volume, and D5 is the minimum dose in 5% of PTV volume. Conformity index (CI) was calculated using [15,16] CI = V95/VPTV 95, where V95 is the treated volume receiving ≥95% dose (reference isodose volume), and PTV volume is the total volume of the PTV.

Statistical analysis

Statistical analysis was done using SPSS Statistics version 23 (IBM Corp., Armonk, NY, USA). Each variable was analyzed for normal distribution. Continuous parametric data were reported as mean +/- SD, while non-parametric data were presented as median (with interquartile range). Paired t-tests for normally distributed data and the Wilcoxon signed-rank test for non-normal data were applied to compare the quantitative data. A p-value of 0.05 or less was taken as statistically significant.

## Results

Table 1 features the demographic and tumor characteristics of the 15 patients (seven males and eight females) included in the study. The mean age was 41.4 (range 18 to 62 years). Around 73.33% of the gliomas were located on the right side, and the mean PTV volume was 271.71 cc. 

**Table 1:** Demographic and clinical characteristics of the high-grade glioma patients included in the study The majority of the tumors were on the right side of the brain in the frontoparietal region. PTV: Planning target volume

Characteristics		Values
Age (years)	Mean	41.4 (+/- 12.01)
	Range	18-62
Sex	Male	7
	Female	8
Grade	III	7
	IV	8
Laterality	Right	11
	Left	4
Location	Frontal	6
	Parietal	3
	Temporal	2
	Parieto-occipital	3
	Parieto-temporal	1
PTV volume (cm3)	Mean (+/- SD)	271.71 (+/- 121.56)
	Median	264.35
Brain PTV volume (cm3)	Mean (+/- SD)	976.59 (154.92)
	Median	991.79

PTV doses

**Figure 1:**
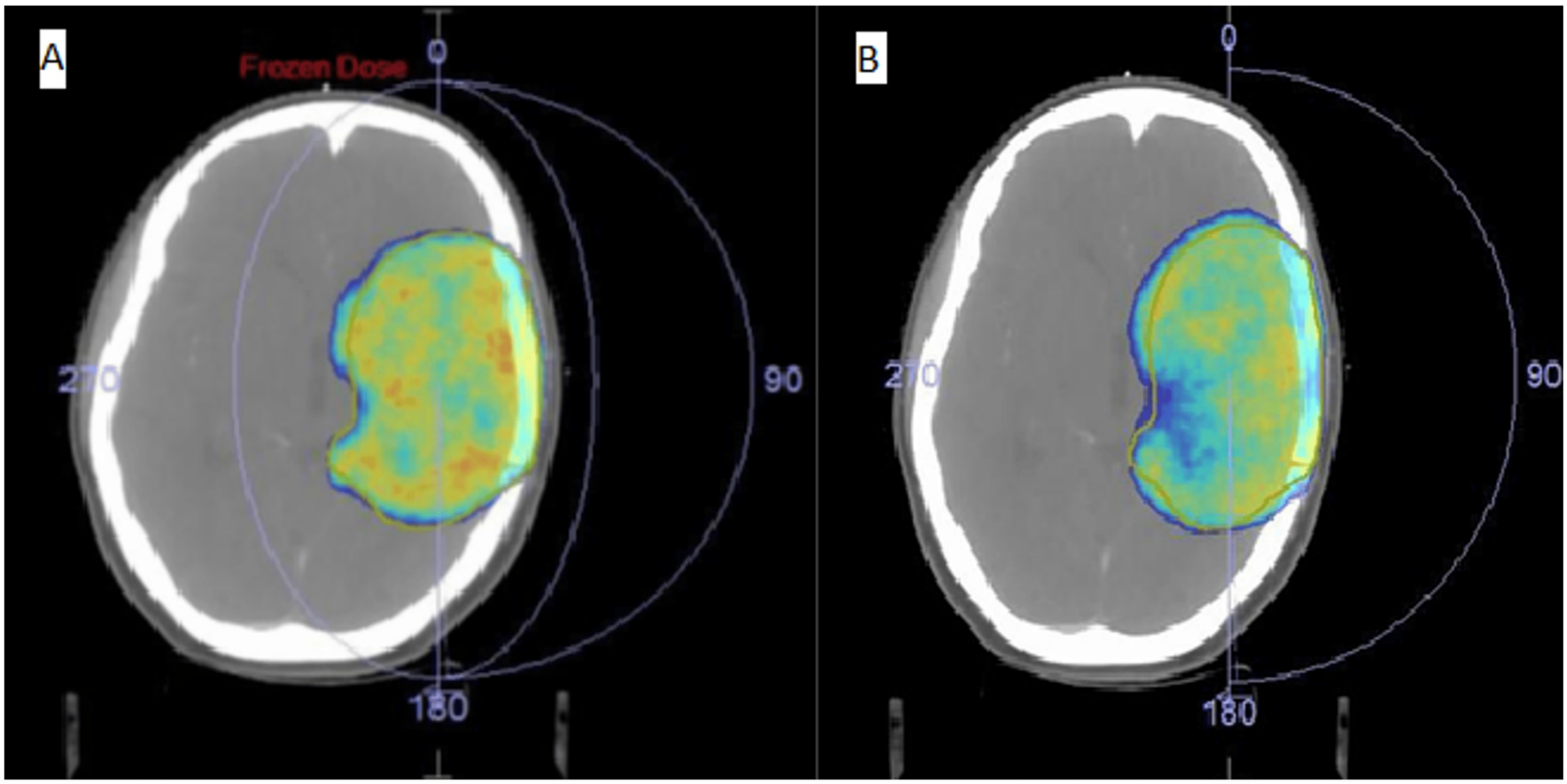
Axial slice of dose colour wash of a representative plan (A: NcVMAT, B: cVMAT) The dose colour wash shows volume receiving a minimum of 95% of the prescribed dose (60Gy/30 fractions). NcVMAT: Noncoplanar volumetric-modulated arc therapy, cVMAT: Coplanar volumetric-modulated arc therapy

The PTV Dmax (NcVMAT 6366.51, cVMAT 6412.65, p = 0.274), PTV Dmean (NcVMAT 6006.31, cVMAT 6048.60, p = 0.86), and PTV minimum dose (Dmin) (NcVMAT 5201.58, cVMAT 5177.30, p = 0.70) did not significantly differ between the two plans (Table 2). A small but insignificant difference was observed between CI (NcVMAT 0.986, cVMAT 0.988, p = 0.12) and HI (NcVMAT 0.948, cVMAT 0.952, p = 0.27) (Table 2). Figure 1 depicts the dose color wash, representing both plans so as to be comparable in terms of target coverage. 

**Table 2:** Comparison of PTV dosimetric parameters The results show a non-significant difference between the two plans. Both CI and HI also had comparable values. PTV: Planning target volume, CI: Conformity index, HI: Homogeneity index, Dmean: Mean dose, Dmax: Maximum dose, Dmin: Minimum dose

Parameters	NcVMAT	cVMAT	p-value
PTV Dmean (cGy)	6006.31	6048.60	0.86
PTV Dmax (cGy)	6366.51	6412.65	0.27
PTV Dmin (cGy)	5201.58	5177.30	0.70
CI	0.9861	0.9883	0.119
HI	0.9484	0.9523	0.27

OAR doses

Table 3 shows a comparison of OAR doses (in cGy) in the two plans, and it was seen that the NcVMAT plan could significantly reduce the maximum dose to the optic chiasm (NcVMAT 2085.7, cVMAT 2281.0, p = 0.002) and the ipsilateral cochlea (NcVMAT 324, cVMAT 1178, p = 0.016). Mean doses to the ipsilateral cochlea (NcVMAT-280.0, cVMAT-882.0, p = 0.005), lens (NcVMAT 335.73, cVMAT 584.07, p = 0.038), and optic nerve (NcVMAT 1321.93, cVMAT 1550.33, p = 0.04) were also significantly lower in NcVMAT plans. The monitor unit (MU) per fraction was 498.93 in the NcVMAT plan, which was insignificantly different compared to 561.93 in the cVMAT plan (p = 0.082) (Table 3).

**Table 3:** The OAR doses and MU comparison between the two plans show better doses for optic chiasma and cochlea with NcVMAT compared to cVMAT with relatively fewer MUs per fraction. NcVMAT: Noncoplanar volumetric-modulated arc therapy, cVMAT: Coplanar volumetric-modulated arc therapy, OAR: Organs at risk, PTV: Planning target volume, MU: Monitor unit, Dmax: Maximum dose, Dmean: Mean dose

OAR		NcVMAT	cVMAT	p-value
Brainstem	Dmax (cGy)	4807.2	4767.1	0.06
	Dmean (cGy)	1699.40	1777.47	0.60
Optic chiasma	Dmax (cGy)	2085.7	2281.0	0.002
	Dmean (cGy)	2172.0	2279.47	0.63
Ipsilateral optic nerve	Dmax (cGy)	2021.53	2277.13	0.07
	Dmean (cGy)	1321.93	1550.33	0.04
Contralateral optic nerve	Dmax (cGy)	1190.27	1273.80	0.66
	Dmean (cGy)	847.40	863.93	0.92
Ipsilateral eyeball	Dmax (cGy)	1374.67	1710.13	0.07
	Dmean (cGy)	566.67	826.33	0.055
Contralateral eyeball	Dmax (cGy)	917.40	930.27	0.95
	Dmean (cGy)	258.0	232.0	0.91
Ipsilateral lens	Dmax (cGy)	477.20	735.60	0.059
	Dmean (cGy)	335.73	584.07	0.038
Contralateral lens	Dmax (cGy)	240.0	215.0	0.82
	Dmean (cGy)	207.0	185.0	0.86
Ipsilateral cochlea	Dmax (cGy)	324.0	1178.0	0.016
	Dmean (cGy)	280.0	882.0	0.005
Contralateral cochlea	Dmax (cGy)	352.0	443.0	0.91
	Dmean (cGy)	302.0	386.0	0.65
Contralateral temporal lobe	Dmax (cGy)	3048.73	3007.93	0.92
	Dmean (cGy)	1598.60	1281.40	0.15
Normal brain (Brain PTV)	Dmax (cGy)	6249	6198	0.69
	Dmean (cGy)	2202	1847.13	0.086
MUs		498.93	561.93	0.082

## Discussion

Treatment of high-grade glioma involves maximal surgically safe resection followed by adjuvant radiation therapy with concurrent and adjuvant temozolomide [3]. The brain is a vital organ with many critical structures; hence, conformal planning is essential to spare these structures. High doses to the PTV and as low as possible to the OAR are desired per the 'as low as reasonably achievable' (ALARA) principle [17]. The 3D conformal radiotherapy (3D-CRT) and IMRT are the current conformal treatment modalities. Different methods for delivery of IMRT treatment include fixed-field IMRT, dynamic IMRT, and VMAT. The VMAT involves single or multiple arcs (partial or complete) delivered in the coplanar or non-coplanar mode for improving conformity. Treatment with non-coplanar beams involves several fixed or rotating radiation beams whose geometric plane is not the same as that of the patient to reduce the beam overlap away from the tumor, as it may improve dose distributions compared to a cVMAT [18].

The VMAT, compared to IMRT, provides equal or sometimes better coverage of PTV with better sparing of the OAR, fewer MUs per fraction, and less time for treatment in case of high-grade glioma [19]. On the flip side, VMAT plans require longer plan optimization times and come with higher integral doses. A study by Sheu et al. had retrospectively reviewed the clinical impact of the dosimetric advantage of VMAT over IMRT in glioblastoma patients. They showed that VMAT resulted in similar oncologic and toxicity outcomes with reduced treatment time compared to IMRT, implying that VMAT may improve resource utilization. Volumetric-modulated arc therapy was advised to be considered as a potential radiation modality for patients with GBM [20].

In the present study, we compared the cVMAT with the NcVMAT technique in high-grade glioma patients. The results of this study show that both techniques are comparable in terms of CI, HI, and PTV Dmax, Dmin, and Dmean between the two plans. Similar results were seen in studies for glioblastoma patients by Hou et al. and for sinonasal tumors by Zhong-Hua et al., where both CI and HI did not differ statistically between cVMAT and NcVMAT [10,21]. However, we cannot directly compare these studies with ours, as the method used for calculation of HI and CI by these researchers was different from that used by us. In both these studies, PTV Dmax, Dmean, and Dmin also did not vary significantly between the two plans. Hou et al. also compared IMRT with cVMAT and NcVMAT plans and found them to have similar CI, HI, and PTV coverage. Contrary to this, Zhong-Hua et al. showed cVMAT and NcVMAT to have better conformity and homogeneity indices compared to coplanar IMRT [10].

In our study we found that the NcVMAT plan is superior compared to cVMAT for the Dmax and Dmean to the ipsilateral cochlea, Dmax to the optic chiasma, and Dmean to the ipsilateral lens and optic nerve. These findings are in line with the findings of Jeong et al., who compared these two plans for nasal cavity and paranasal sinus cancer, but Hou et al., in their dosimetric study on glioblastoma patients, showed a non-significant difference between NcVMAT and cVMAT techniques in terms of doses to the OAR [5,21].

Hou et al. also showed that the VMAT plans require fewer MUs compared to IMRT plans but could not find a significant difference between cMAT and NcVMAT plans [21]. In our study too, we found that NcVMAT plans showed a statistically insignificant difference in MUs per fraction compared to cVMAT. This indicates almost equal beams with regard to treatment time in cVMAT compared to NcVMAT. But, since non-coplanar treatment delivery involves manual couch movement, the on-couch time in non-coplanar treatment is longer compared to coplanar treatment. Since it was a dosimetric study, we could not access the on-couch time for these patients. A new solution with specialized functions for developing isocentric VMAT plans for non-coplanar MLC-based treatment with minimal workload, including automated location of the isocenter, automated optimization, non-coplanar beam arrangement, and delivery, could be an answer [22,23]. Such a delivery technique has shown significantly better dose distribution and better sparing of OAR for brain lesions [24,25]. Our study showed both techniques resulted in clinically acceptable plans in terms of target coverage. As far as OAR sparing is concerned, the non-coplanar plans have an upper hand in sparing OAR, especially optic structures, which is in line with the previously published studies.

Limitations

The current study has a few limitations. The sample size is small, the dosimetric accuracy of NcVMAT is not validated, there is no treatment time comparison, there is variable location of tumors (majority in the frontal lobe), and the clinical implications of this dosimetric study (e.g., toxicity reduction or neurocognitive preservation) were not evaluated. Despite these limitations, our study provides an important contribution regarding the delivery of adequate doses to the target and minimizing doses to the OAR.

## Conclusions

Our study shows that NcVMAT and cVMAT plans have comparable CI, HI, and PTV coverage, while NcVMAT plans demonstrated a notable reduction in point doses to the optic chiasma and cochlea, with a similar number of monitor units per fraction. These dosimetric findings suggest that NcVMAT may offer certain advantages in terms of OAR sparing without compromising target coverage. While these results are encouraging, they should be interpreted with caution given the inherent limitations of a single-institution planning study. Noncoplanar volumetric-modulated arc therapy could be considered as a potentially preferable technique for the treatment of high-grade gliomas, but further validation through larger, prospective clinical studies would be valuable before any definitive clinical recommendations can be made.
